# Antibacterial potential of *Forsythia suspensa* polysaccharide against resistant *Enterobacter cloacae* with SHV‐12 extended‐spectrum β‐lactamase (ESBL)

**DOI:** 10.1111/jcmm.15510

**Published:** 2020-06-25

**Authors:** Jun Liu, Liyao Lin, Zhen Jia, Jing Chen, Zuguo Zhao, Yi Zhao, Zhujin Xu, Zhen Guo

**Affiliations:** ^1^ Laboratory of Pathogenic Biology Guangdong Medical University Zhanjiang China; ^2^ Department of Cardiothoracic Surgery Affiliated Hospital of Guangdong Medical University Zhanjiang China; ^3^ Department of Neurology Affiliated Hospital of Guangdong Medical University Zhanjiang China; ^4^ Geriatrics Center Affiliated Hospital of Guangdong Medical University Zhanjiang China

**Keywords:** antibacterial activity, *Enterobacter cloacae* producing SHV‐12 ESBL, *Forsythia suspense* polysaccharide

## Abstract

In this study, a homogenous polysaccharide (FSP), with an average molecular weight of 9.08 × 10^4^ Da, was isolated from *Forsythia suspense* and its antibacterial potential against *Enterobacter cloacae* producing SHV‐12 ESBL was investigated. Growth kinetics, in vitro competition and biofilm formation experiments demonstrated that SHV‐12 ESBL contributed to a fitness benefit to *E cloacae* strain. The antibacterial activity of FSP (2.5, 5.0 and 10.0 μg/mL) was tested against *E cloacae* bearing SHV‐12 ESBL gene using bacterial sensitivity, agar bioassay and agar well diffusion assays. It was found that the addition of FSP demonstrated potent antibacterial activities against this bacterial as showed by the decrease of bacterial growth and the increase of the inhibition zone diameter. Furthermore, SHV‐12 ESBL gene expression was decreased in *E cloacae* strain following different FSP treatment in a concentration‐dependent manner. In conclusion, these data showed that FSP exhibited potent good antibacterial activity against *E cloacae* producing SHV‐12 ESBL via inhibition of SHV‐12 ESBL gene expression, which may promote the development of novel natural antibacterial agents to treat infections caused by this drug‐resistant bacterial pathogen.

## 1 INTRODUCTION

In the past decades, the *Enterobacter cloacae* family (*Enterobacter hormaechei*, *Enterobacter asburiae*, *Enterobacter nimipressuralis*, *E. cloacae*, *Enterobacter ludwigii* and *Enterobacter kobei*) has attracted more and more attention, due to its prevalence as nosocomial pathogens, predominately in intensive care units (ICUs). Among them, *E cloacae* is classified as a well‐recognized nosocomial Gram‐negative pathogen, responsible for a wide range of infections, including bacteraemia, soft tissue, lower respiratory tract and urinary tract infections, as well as intra‐abdominal infections.^1^, ^2^ With the ability to produce SHV‑type extended‑spectrum β‐lactamases (ESBLs) in this pathogenic microorganism, *E cloacae* has become more insensitive to β‑lactam antibiotics.^3^, ^4^ In different kind of SHV‑type ESBLs, the SHV‑12‑type ESBL is widely reckoned as the most popular one in Asia.^5^ In China, the infection rate of *E. cloaca*e producing SHV‐12 ESBL ranked the third place merely behind *Escherichia coli* and *Klebsiella pneumoniae* among enterobacteriaceae.^6^ Additionally, increasing use of broad‐spectrum antimicrobial agents has resulted in the high incidence of SHV‑12‑type ESBL *E cloaca*e at an alarming rate.^7^ The development of such extensive multidrug resistance and a lack of corresponding enzymatic inhibitors have restricted the choice of antibiotics in clinical use, and therefore, the design of new antibiotics is the ideal approach to overcome this problem.

Traditional Chinese medicine (TCM) has been recognized as a rich resource of antimicrobial drugs for thousands of years in China.^8^, ^9^ *Forsythia suspensa* (Thunb.) Vahl. is widely distributed in Asia including China and many European countries, and its fruit is a famous TCM, known as ‘Lianqiao’ in Chinese with the functions of antioxidation, cardioprotection, anti‐inflammation, liver protection and antifatigue.^10^, ^11^ More importantly, it is also well known for their remarkable and wide range antimicrobial activity against bacteria, fungi and viruses.^12^, ^13^ To date, a large amount of ingredients, such as phenylethanoid glycosides, volatile oils, flavonoids, terpenes and lignans, had been verified from *F suspensa*.^14^ Although a variety of pharmacological activities of *F suspensa* and its constituents have been reported, no information is available about the polysaccharides and its antibacterial activity from this plant. Currently, the role of SHV‑12 ESBL in the regulation of the fitness of *E cloacae* has not been carried out, which are closely related to the antibiotic resistance. Therefore, we aim to isolate the polysaccharide from *F suspense* and examine its antibacterial activity. Furthermore, we clarify if the SHV‑12 ESBL is involved in the fitness change of *E cloaca* to explore its mechanism of action.

## 2 MATERIALS AND METHODS

### 2.1 Materials and chemicals

*F suspensa* was obtained from Hebei Guang Ming Prepared Medicinal Herbs Co., Ltd. DEAE Sepharose Fast Flow and Sepharose CL‐6B Fast Flow were from Amersham. Standard T‐series Dextran (668 000, 410 000, 273 000, 148 000, 48 600, 23 800, 11 600, 5200 Da) and monosaccharide standards (rhamnose, galactose, xylose, mannose, glucose, arabinose, glucuronic acid, galacturonic acid) were obtained from Sigma Chemical Co. TRIzolR Reagent was from Invitrogen, and qPCR Kit was from QIAGEN. All the other chemicals used were of analytical grade and were purchased from Sinopharm Chemical Reagent Co. Ltd.

### 2.2 Isolation and purification of polysaccharide

The fruit of *F suspensa* (0.5 kg) was mechanically chopped into small pieces and preliminarily treated with 95% ethanol (6000 mL) there times and 3 hours each time at 90°C to remove low molecular components, including oligosaccharide, lipids and pigments in the samples. The residue was further immersed in boiling water (8000 mL) for three times and each time for 2 hours. Then, the extract was filtered and collected by centrifugation (1700 × *g*, 10 minutes) at room temperature. All supernatant were collected, concentrated and precipitated with adding 4 volumes of 95% ethanol at 4°C for 24 hours. The precipitate was subsequently re‐dissolved in distilled water and deproteinized five times by Sevag reagent (chloroform: butanol 4:1, v/v). After removing Sevag reagent, the remaining water layer was concentrated and added with 5 volumes of 95% ethanol (v/v) to precipitate the crud polysaccharide (CFSP, 22.7 g).

Each aliquot of 80 mg crude polysaccharides was added with 5 mL deionized water, centrifuged (1700 × *g*, 10 minutes) and filtered by a 0.45 μm membrane. The resulting filtrate was loaded onto a column (2.0 cm × 40 cm, Cl^−^ form) of DEAE Sepharose Fast Flow and eluted with distilled water and a stepwise gradient of NaCl aqueous solution (0.2, 0.5 and 1 mol/L) at a flow rate of 4.0 mL/min to yield FSP1, FSP2, FSP3 and FSP4, respectively. Fraction with of 8 mL in each tube was collected using an automated fraction collector and monitored by phenol‐sulphuric acid method at 490 nm.^15^ Subsequently, the main fraction FSP1 eluted with distilled water was collected for further purification by gel permeation chromatography on a Sepharose CL‐6B Fast Flow (2.6 cm × 100 cm) column with 0.1 mol/L NaCl as the mobile phase at a flow rate of 2.0 mL/min, affording only one purified fraction (FSP).

### 2.3 Chemical composition, monosaccharide analysis, homogeneity and molecular weight determination

The total carbohydrate content of polysaccharide sample was measured by the phenol‐H_2_SO_4_ method, with glucose as the standard.^15^ Uronic acid content was determined according to a m‐hydroxydiphenyl colorimetric method by using d‐galacturonic acid as the standard.^16^ In addition, protein content in the polysaccharide fraction was measured as previously described by Bradford's method^17^ using bovine serum albumin (BSA) as the standard.

Monosaccharide composition was determined using gas chromatograph (GC) as described by Liu et al^18^ In short, the polysaccharide samples were hydrolysed with 4 mL of 2 mol/L TFA at 115°C for 2.5 hours in a sealed test tube. After excessive TFA was completely removed, the hydrolysates were mixed with 10 mg of NaBH_4_ for 3 hours at room temperature and supplemented with each 0.5 mL of anhydride and pyridine for 1 hour at 60°C to be converted into the alditol acetates before GC analysis.

The polysaccharide homogeneity and its molecular weight evaluation were determined by high‐performance gel filtration chromatography (HPGPC) on an Agilent1100 HPLC instrument, matched with an Agilent RID‐10A refractive index detector and TSK‐GEL G4000SW column (7.8 mm × 300 mm). The samples were dissolved in 0.1 mol/L NaNO_3_ and filtered (0.45 μm) before injection. The flow rate was 0.5 mL/min at 40°C, with 1.6 mPA. The molecular mass was estimated by reference to a calibration curve made from a set of Dextran T‐series standards.

### 2.4 Bacterial strains and growth conditions

The clinical isolates used were two strains of *E cloacae*: one is SHV‐12 ESBL‐negative and another is SHV‐12 ESBL‐positive strain. Both of them were kept and identified using RT‐PCR in our laboratory of Pathogenic Biology, Guangdong Medical University.

### 2.5 The role of SHV‐12 ESBL on fitness of drug‐resistant *E cloacae*

#### 2.5.1 Growth curve assays

To assess in vitro fitness costs under noncompetitive conditions, the growth kinetics of *E cloacae* strain with or without SHV‐12 ESBL were constructed as described previously^19^ in microplates coupled to a Multiscan spectrophotometer (Thermo Scientific). Growth curves were made by diluting ~2 × 10^4^ bacteria into 200 μL of LB broth in a 96‐well microplate, which was kept at 37°C with constant shaking. Absorbance was measured at 600 nm at 60‐min interval post‐dilution. Each curve was performed three times in the same microplate.

#### 2.5.2 In vitro competition assays

This competition assay was performed between SHV‐12 ESBL*‐*positive and SHV‐12 ESBL‐negative *E cloacae* strains. Briefly, two bacterial strains from LB broth cultures during exponential growth were mixed at an initial ratio of 1:1 and grown for 12 hours (about 20 generations). Approximately 10^3^ cells from each of the mixtures were inoculated into 10 mL of LB broth and grown at 37°C with shaking (180 rpm) for 16 to 18 hours. The mixed culture was transferred to LB agar alone or LB agar containing 50 μg/mL of colistin by 10‐fold serial dilution and incubated for 24 hours at 37°C to determine the total colony‐forming units (CFU) of each mixture and the CFU of *E cloacae* showing SHV‐12 ESBL, respectively, The competition index (CI) represents as the ratio of the CFU of SHV‐12 ESBL*‐*positive *E cloacae* to the CFU of the parental strain. By definition, CI = 1 means that SHV‐12 ESBL‐positive *E cloacae* strain has no fitness effect. CI value higher than 1 or lower than 1 indicates increased or decreased fitness, respectively.^20^ The relative fitness cost of SHV‐12 ESBL*‐*positive *E cloacae* was compared with that of SHV‐12 ESBL*‐*negative *E cloacae* strain. Each experiment was performed in duplicate and repeated four times independently to calculate the median values for each CI.

#### 2.5.3 Biofilm assay

Biofilm formation assay under static conditions was carried out as described previously, with minor modifications.^21^ Briefly, two strains were grown for 24 hours at 37°C in TSB medium and overnight cultures were diluted 1:100 in TSB medium supplemented with 0.5% glucose. Then, 200 μL of diluted cell suspension was transferred into flat‐bottom 96‐well plates and incubated overnight at the room temperature without shaking. Some wells filled with fresh TSB were used as blank controls. At the end, unattached cells were carefully wiped off with gently phosphate‐buffered saline (PBS) washing for three times. Subsequently, the plates were air‐dried, stained with 200 μL of crystal violet (0.5%) for 15 minutes, and washed to remove the unbound stain. After the plates were air‐dried for 2 hours, 200 μL of dimethyl sulfoxide (DMSO) was seeded into the wells to dissolve the attached dying material and the absorbance was measured at 600 nm. The assays were performed for three independent times.

### 2.6 In vitro antibacterial analysis

#### 2.6.1 Sensitivity assay

For determining the susceptibility of SHV‐12 ESBL*‐*positive *E cloacae* strains to FSP, 1 mL of LB at pH 7.4 was inoculated with a single clone and cultured to logarithmic growth phase. After that, 300 μL of the culture was added into pre‐warmed fresh LB broth (30 mL) for 24 hours. Thereafter, different growth of bacteria was assessed per hour through determining the optical density of the culture at 600 nm using the Lambda 25 UV–visible spectrophotometer. The culture without FSP was used as control, and levofloxacin (0.5 μg/mL) was used as antibacterial positive control. All the experiments were determined at least three independent tests.

#### 2.6.2 Agar bioassay

The effect of FSP on SHV‐12 ESBL*‐*positive and SHV‐12 ESBL*‐*negative *E cloacae* strains was investigated by culturing the bacteria on LB agar plates (10^5^ CFU of each strain per plate) supplemented with FSP (2.5, 5 and 10 μg/mL). Plate without strains was defined as a control. Thereafter, the plates were subject to incubation at 37°C for 12 hours and the number of colonies was counted.

#### 2.6.3 Agar well diffusion assay

The antibacterial activity of FSP was also confirmed by agar well diffusion assay,^22^ with slight modification. In brief, *E cloacae* strains (10^5^ CFU/mL) at exponential growth phase were poured uniformly into LB agar plates supplemented with different concentrations (2.5, 5 and 10 μg/m) of FSP and then cultured at 37°C for 12 hours. The same amount of sterile water was taken as the control, and levofloxacin (2.5 μg/disc) was used as antibacterial positive control. Following treatment, the clear zones were taken as inhibitory zones and its diameters were determined in millimetres.^23^

### 2.7 RNA extraction and quantitative real‐time PCR (qRT‐PCR) analysis

The expression levels of the SHV‐12 ESBL genes were determined by qRT‐PCR. For RNA extraction, bacteria were grown aerobically in LB broth until mid‐log phase. The culture without FSP was used as control, and levofloxacin (0.5 μg/mL) was used as antibacterial positive control. Next day, total RNA was collected using TRIzolR Reagent as per the manufacturer's instructions, and total RNA concentrations were quantified spectrophotometrically using a UV–visible NanoDrop 2000 spectrophotometer. cDNA was synthesized from 2 μg of total RNA using QIAGEN qPCR Kit and was used for qRT‐PCR assay with specific primers (forward primer: 5′‐GGT TAT GCG TTA TAT TCGCC‐3′and reverse primer: 5′‐TTA GCG TTG CCA GTG CTC‐3) to examine the SHV‐12 ESBL level. The comparative CT method was applied to assess the relative level of mRNA. For relative quantification, the level of SHV‐12 ESBL was expressed as –fold changes normalized to the Ct value of GAPDH. All assays were carried out in triplicates to ensure reproducibility.

### 2.8 Statistical analysis

All results are the means of 3 experiments ± standard error (SE). Statistical analysis was performed using Student's *t* test or ANOVA using GraphPad software package for Windows (Prism version 3.00). A value of <0.05 was considered statistically significant.

## 3 RESULTS

### 3.1 Isolation, purification and characterization of polysaccharide

**FIGURE 1:**
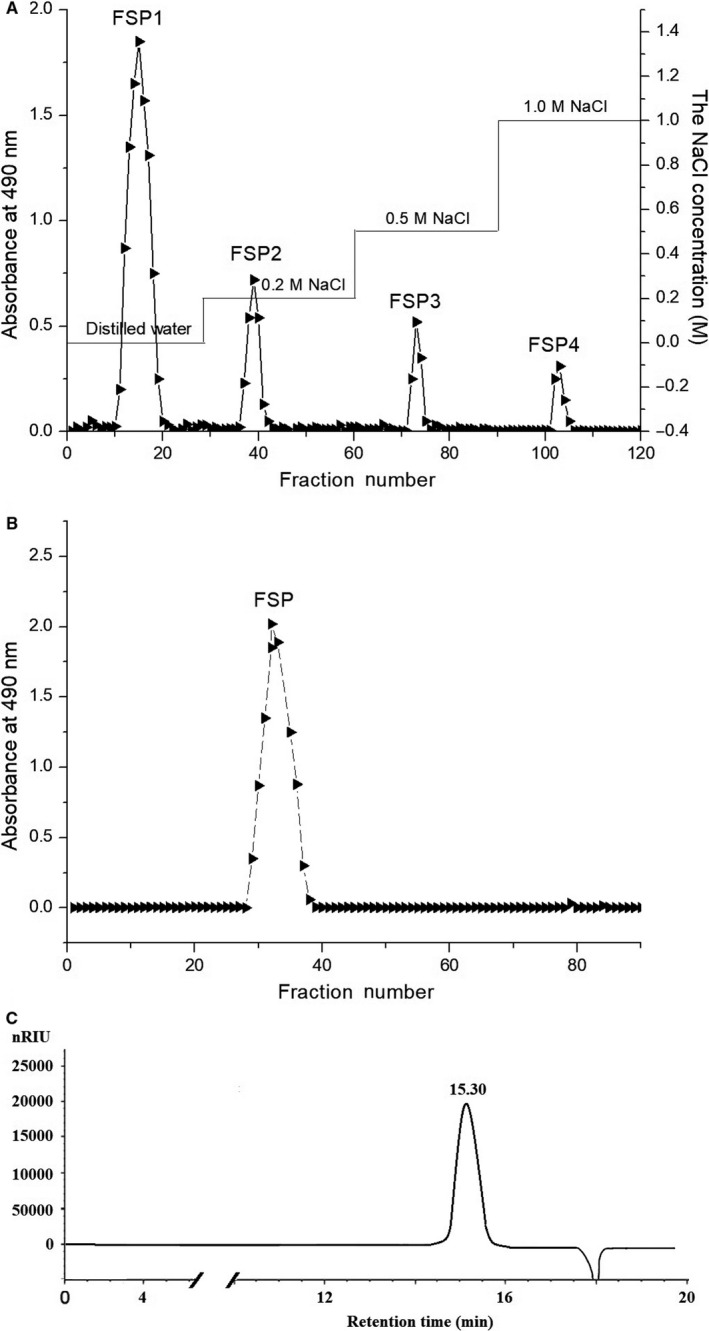
Isolation, purification and HPGPC profile of the polysaccharide. A, Elution profile of the CFSP from *Forsythia suspensa* on DEAE sepharose fast flow anion‐exchange chromatography column. B, Elution profile of the FSP1 on Sepharose CL‐6B fast flow gel permeation chromatography column. (C) Chromatogram of FSP on HPGPC

The polysaccharide CFSP, with a yield of 4.54% of the starting raw material, was isolated from defatted fruit of *F suspensa* by hot‐water extraction, ethanol precipitation, deproteinization and lyophilization. Total CFSP was first fractioned through a DEAE Sepharose Fast Flow anion‐exchange chromatography column according to their different ionic groups. Four fractions, FSP1, FSP2, FSP3 and FSP4, were obtained by stepwise elution with distilled water, 0.2, 0.5 and 1 mol/L NaCl, respectively (Figure 1A). Following this, the neutral fraction FSP1 was further purified on a column of Sepharose CL‐6B Fast Flow (Figure 1B), giving a purified polysaccharide FSP (0.53% of the raw material). The total carbohydrate content was 97.8% based on the phenol‐sulphuric acid method. The absence of absorbance at 280 and 260 nm in the UV spectrum indicated no protein and nucleic acid in the polysaccharide. The chromatogram of FSP in HPGPC presented a single, symmetrical and narrow elution peak, confirming its homogeneity (Figure 1C). The average molecular weight of FSP was estimated to be 9.08 × 10^4^ Da on the basis of calibration with standard D‐series dextrans (LogMw = −0.3025*t* + 9.5862; *R* = 0.998, *t* = 15.3 min). GC analysis of the monosaccharide composition indicated that FSP was mainly composed of one kind of monosaccharide, namely glucose.

### 3.2 Growth kinetics, in vitro competition and biofilm formation experiments of *E cloacae* strain with or without SHV‐12 ESBL

**FIGURE 2:**
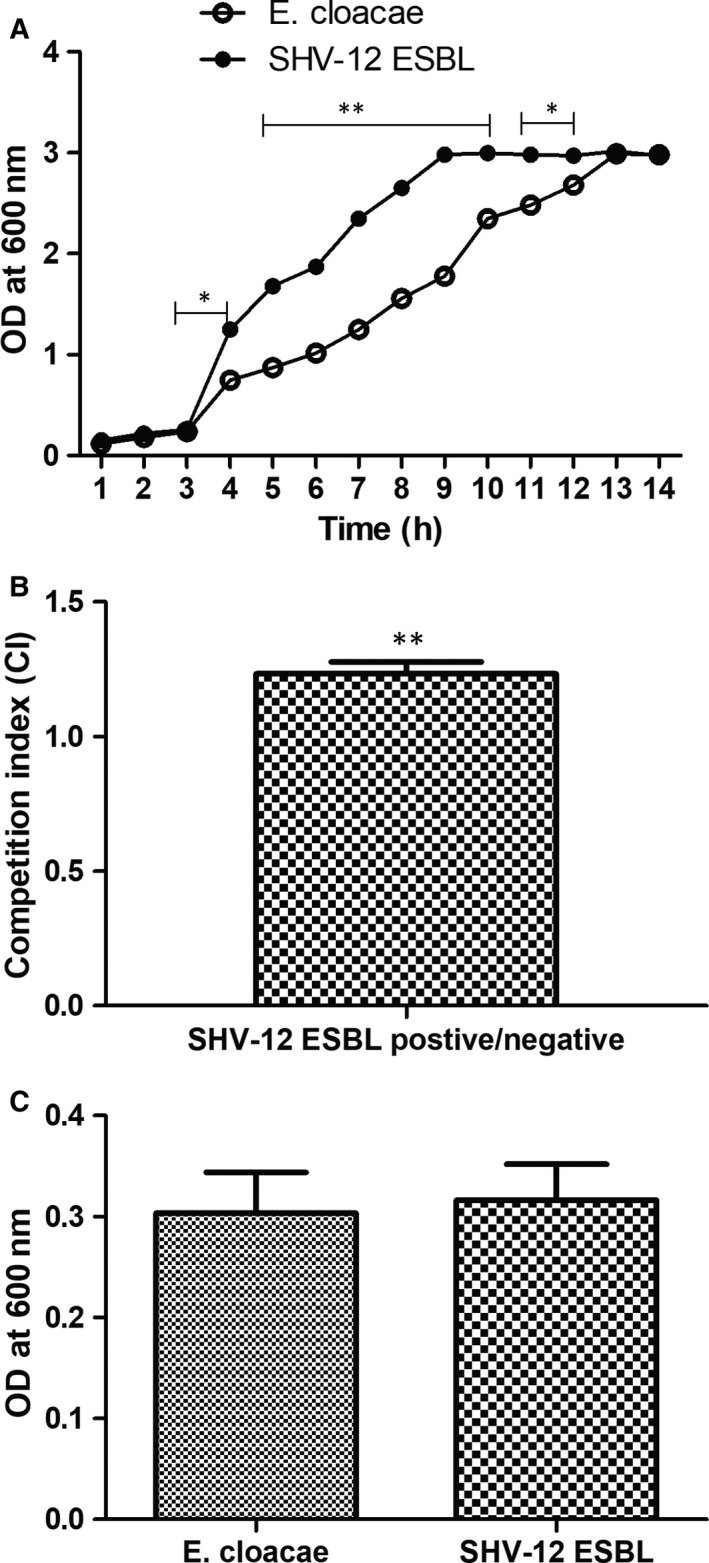
The role of SHV‐12 ESBL on fitness of drug‐resistant *Enterobacter cloacae*. A, Comparison of the growth kinetics of *E cloacae* strain with or without SHV‐12 ESBL. B, In vitro growth competition curves of *E cloacae* strain with or without SHV‐12 ESBL. C, Biofilm formation of *E cloacae* strain with or without SHV‐12 ESBL. The mean ± SE for three replicates are illustrated. ^*^ *P* < .05, ^**^ *P* < .01 vs control

To assess whether SHV‐12 ESBL contributed a fitness defect to *E cloacae* strain, growth curves, in vitro competition and biofilm formation experiments were performed under the same culture conditions, as described in Materials and Methods. As shown in Figure 2A, the growth rate of *E cloacae* strain was not substantially affected by the presence of SHV‐12 ESBL. The time bacterial growth of *E. cloaca*e reached logarithmic growth stage and became saturated was later than that in resistant mutants containing SHV‑12‑type ESBL. Consistent results were obtained in competition experiments, and the CI values greater than 1 indicated that *E. cloaca*e producing SHV‑12‑type ESBL showed significant fitness benefit when co‐inoculated with the *E. cloaca*e strain without SHV‑12‑type ESBL (Figure 2B). Figure 2C depicted the results of biofilm formation assays for SHV‐12 ESBL‐positive and SHV‐12 ESBL‐negative *E cloacae* strain. Biofilm‐forming activity of *E. cloaca*e control was lower than that of *E. cloaca*e strain producing SHV‑12‑type ESBL, but the difference in biofilm formation was not statistically significant.

### 3.3 Antibacterial activity of FSP on E cloacae strain with SHV‐12 ESBL

**FIGURE 3:**
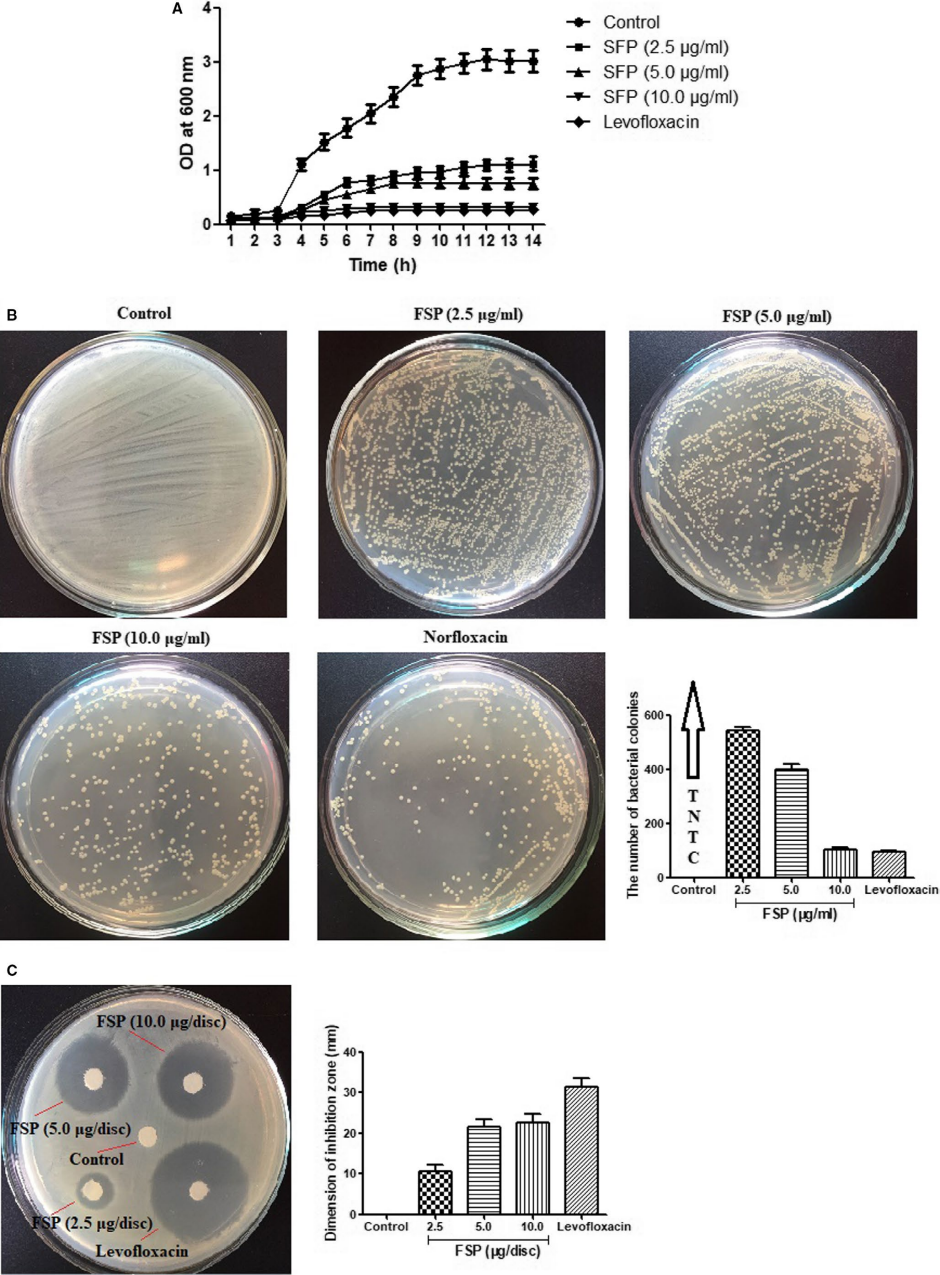
In vitro antibacterial analysis of the polysaccharide. A, Bacterial growth curve of SHV‐12 ESBL‐positive *Enterobacter cloacae* in LB media in the presence of FSP (2.5, 5.0 and 10.0 μg/mL). B, Agar bioassay for SHV‐12 ESBL‐positive *E cloacae* in the presence of FSP (2.5, 5.0 and 10.0 μg/mL). C, Agar well diffusion assay for SHV‐12 ESBL‐positive *E cloacae* in the presence of FSP (2.5, 5.0 and 10.0 μg/mL). The mean ± SE for three replicates are illustrated

The growth curve of *E cloacae* strain with SHV‐12 ESBL in the presence of FSP (2.5, 5.0 and 10.0 μg/mL) was shown in Figure 3A. At the first 3 hours, both the sample of FSP and the control had a lag phase, and then, the control grew in a faster rate with increasing DO value at 600 nm as compared with the OD_600_ value of FSP at each time. Similarly, FSP exhibited a high inhibitory effect on the growth of SHV‐12 ESBL‐positive *E cloacae* strain on LB agar plates in a concentration‐dependent manner at 12 hours (Figure 3B). In addition, the average dimension of inhibition zone resulting from FSP application against SHV‐12 ESBL‐positive *E cloacae* strain ranged from 8.71 ± 0.95 to 30.41 ± 2.54 mm with the increasing concentration (Figure 3C). These results revealed that FSP was more effective against the growth of SHV‐12 ESBL‐positive *E cloacae* strain.

### 3.4 Effect of FSP on SHV‐12 ESBL gene expression

**FIGURE 4:**
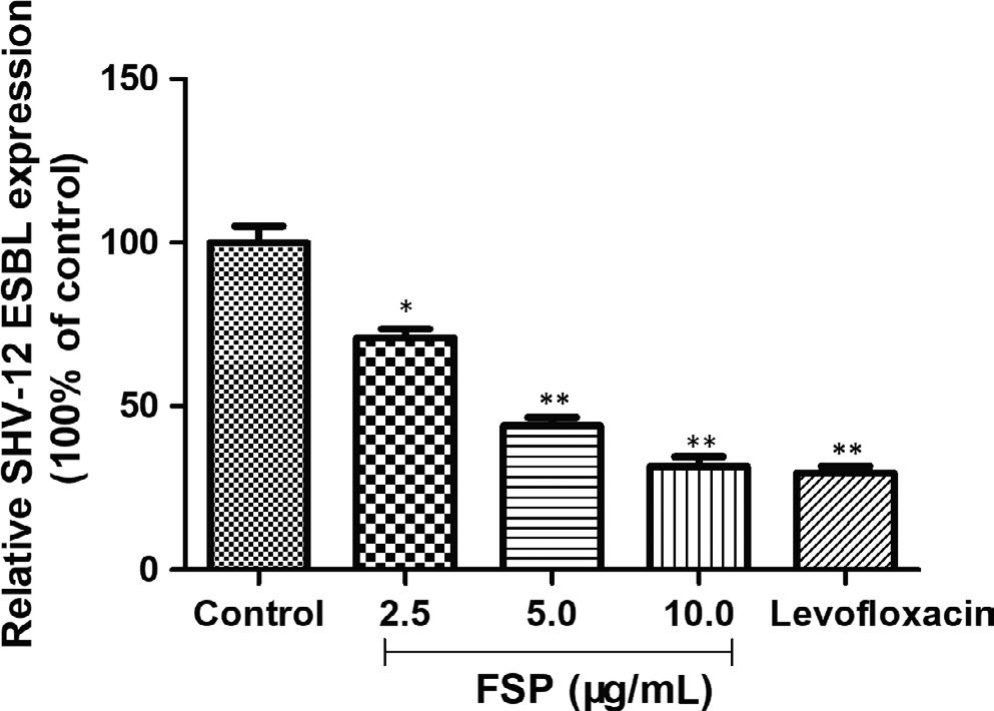
Relative mRNA levels of SHV‐12 ESBL in FSP‐treated *Enterobacter cloacae* strains measured by qRT‐PCR. The mean ± SE for three replicates are illustrated. ^*^ *P* < .05, ^**^ *P* < .01 vs control

The transcript levels of SHV‐12 ESBL were examined in all FSP‐treated *E cloacae* strains. The qRT‐PCR result in Figure 4 showed the dose‐dependent inhibitory effects of FSP on SHV‐12 ESBL gene expression. These results indicated that inhibition of SHV‐12 ESBL gene expression was, at least in part, involved in the antibacterial activity of FSP on *E cloacae* strain with SHV‐12 ESBL in vitro.

## 4 DISCUSSION

Drug resistance with wide spread of antibiotic‑resistant strains is a consequence of the worldwide antibiotic abuse, and this has caused acute challenge for human health.^24^, ^25^ Notably, with the increase of bacterial resistance to antibiotics, effective and nontoxic antibiotics for infections caused by multidrug‐resistant Gram‐negative pathogens become decreasing.^26^ Furthermore, using antibiotics and other synthetic compounds have mountains of adverse effects to human.^27^ Currently, much interest has been focused on searching for novel natural antibacterial agents from TCM that are rich in bioactive substances and well known for their antimicrobial and antibiofilm properties.^28^, ^29^

SHV‐12 ESBL plays a crucial role in *E cloacae* resistance to β‐lactam antibiotics, which can efficiently hydrolyse antibiotics and render them ineffective.^30^ As a result, the treatment option for infection induced by *E cloacae* has become difficult due to the much emergence of SHV‐12 ESBL, causing resistance to antibiotics. First, to verify whether SHV‐12 ESBL exhibits a fitness defect or benefit compared to its parental strain without SHV‐12 ESB, growth curves, in vitro competition and biofilm formation experiments were performed in this study. The growth rates, biofilm formation and in vitro competition experiments of *E cloacae* strains harbouring with SHV‐12 ESBL or not were examined, and significant difference between all these experimental strains was observed. For *E. cloaca*e with SHV‑12‑type ESBL, a slight growth rate increase was observed relative to *E. cloaca*e. Moreover, *E. cloaca*e producing SHV‐12 ESBL had CI values >1 and exhibited large amounts of biofilm formation than *E cloacae* control, demonstrating the fitness benefit under the current condition by SHV‐12 ESB.

Next, with the aim of searching for a potent antibacterial substitute, we isolated and purified one homogenous polysaccharide from defatted fruit of *F suspense*, with an average molecular weight of 9.08 × 10^4^ Da. FSP was a kind of glucan, as identified by GC analysis. To further explore whether *E cloacae* strain with SHV‐12 ESBL was sensitive to FSP, antibacterial activity of FSP was evaluated by sensitivity, agar growth and agar diffusion assay. The control cells grew in a fast rate with increasing DO value at 600 nm, kept stable until end. In contrast, the OD_600_ value kept a slower growth rate than the control at each time point after 3 hours and even remained at a low value till the end. This trend was also observed in agar plates with less bacterial colony in the presence of SPF. Furthermore, FSP showed a high inhibitory effect on SHV‐12 ESBL‐positive *E cloacae* strain with the bigger diameter of inhibition zone than control. These results apparently indicated that *E cloacae* strain with SHV‐12 ESBL was more susceptible to FSP treatment. Consistent with these observations, qRT‐PCR indicated that the inhibitory effect of FSP on SHV‐12 ESBL‐positive *E cloacae* strain was negatively related to the SHV‐12 ESBL gene expression.

## 5 CONCLUSIONS

In conclusion, our present study provided the first evidence that the acquisition of SHV‐12 ESBL promoted the fitness benefit of *E cloacae* strain and FSP possessed a potent antibacterial activity towards SHV‐12 ESBL‐positive *E cloacae* strain, which was achieved by suppressing SHV‐12 ESBL gene expression, suggesting that FSP might be a new natural antibacterial alternative in pharmaceutical industries. Further testing of this plant material in animal models will be required to uncover their suitability for clinical use in humans.

## CONFLICT OF INTEREST

The authors declare that they have no competing interests.

## AUTHOR CONTRIBUTION

**Jun Liu:** Conceptualization (equal); Formal analysis (equal); Investigation (equal); Supervision (equal); Writing‐original draft (equal); Writing‐review & editing (equal). **Liyao Lin:** Conceptualization (equal); Data curation (equal); Formal analysis (equal); Methodology (equal). **Zhen Jia:** Formal analysis (equal); Investigation (equal); Methodology (equal); Validation (equal). **Jing Chen:** Formal analysis (equal); Funding acquisition (equal); Investigation (equal); Methodology (equal); Resources (equal); Software (equal). **Zuguo Zhao:** Investigation (equal); Methodology (equal); Software (equal); Validation (equal); Visualization (equal). **Yi Zhao:** Data curation (equal); Formal analysis (equal); Investigation (equal). **Zhujin Xu:** Data curation (equal); Formal analysis (equal); Investigation (equal). **Zhen Guo:** Data curation (equal); Formal analysis (equal); Methodology (equal).

## AUTHOR CONTRIBUTIONS

LJ and LL designed the experiment and interpreted the results. LJ and JZ drafted the manuscript. JZ, CJ, ZZ, ZY, XZ and GZ performed the experiments. All authors have read and approved the final manuscript.

## Data Availability

All data generated or analysed during this study are included in this article.
